# Parents’ Perception of the Benefit of Receiving a Patient Information Leaflet Prior to Attending a Craniofacial Multidisciplinary Team Appointment

**DOI:** 10.1177/10556656231219579

**Published:** 2023-12-13

**Authors:** Moa Millgård, Anita Myhre, Bernt J. Due-Tønnessen, Kristin J. Billaud Feragen

**Affiliations:** 1 Centre for Rare Disorders, 155272 Oslo University Hospital, Oslo, Norway; 2 Department of Neurosurgery, 155272 Oslo University Hospital, Oslo, Norway

**Keywords:** parental perception, team care, intervention, familial adjustment, craniofacial surgery

## Abstract

**Objective:**

To investigate how a patient information leaflet describing what to expect during a craniofacial multidisciplinary team (MDT) appointment is experienced by parents of children with craniofacial anomalies (CFAs) and whether it helps with preparation for the appointment.

**Design:**

Combination of qualitative and quantitative design.

**Setting:**

Norwegian National Unit for Craniofacial Surgery.

**Participants:**

Thirty-three parents of children with CFAs completed the questionnaire and fourteen were subsequently interviewed.

**Interventions:**

A patient information leaflet, sent to all parents before their MDT appointment.

**Main Outcome Measures:**

Descriptive questionnaire data and interview data.

**Results:**

All parents (*N*  =  33, 100%) found the leaflet easy to understand, while 31 (93.9%) found it provided helpful information. However, many first-time attendees still found the MDT setting overwhelming.

**Conclusions:**

A leaflet may be helpful for parents when preparing for their child's MDT appointment. However, some parents may need additional support and information related to their child's treatment pathway.

## Introduction

Being the parent or primary caregiver (who will henceforth be referred to as ‘parents’) of a child with a craniofacial anomaly (CFA) is often associated with several challenges related to the child's condition.^1–3^ In many cases, treatment is complex and long-term, and it can place a significant psychological burden on both the child and their family.^4–6^

Patients with CFAs are commonly treated by centralised craniofacial multidisciplinary teams (MDTs), which are organised to accommodate the need for coordinated treatment and follow-up.^7,8^ Craniofacial MDTs have been the evaluation and treatment model of choice in various centres internationally.^9^ At Oslo University Hospital, a formally organised team model was set up in the late 1990s for all patients within the health region. In 2002, treatment was centralised at a national level, and the Norwegian National Unit for Craniofacial Surgery was established. Since then, all patients in Norway with CFAs (except clefts) have been evaluated by the national MDT.

The MDT consists of, among others, neurosurgeons, plastic surgeons, dentists, otolaryngologists, ophthalmologists, oral and maxillofacial surgeons, speech and language therapists, geneticists, radiologists, counsellors from Centre for Rare Disorders and MDT coordinators. The objective of the MDT is to examine and follow up all children with CFAs and to develop treatment plans that can be implemented in coordination with local healthcare providers. Patients are invited to an outpatient appointment that lasts for approximately 20–30 min, where around 15 specialists are present and contribute or ask questions of the patient/parent when required. The frequency of follow-up appointments depends on the patient's needs and the complexity of their condition.

The consultation room has a seating arrangement similar to a classroom layout, with members of the MDT being seated in rows facing the patient. Parents or other accompanying persons sit next to the patient. One of the MDT members sits beside them and leads the conversation. During the consultation, one or more specialists may examine the patient.

The consultation itself and the MDT setting can be experienced as emotionally demanding and overwhelming by both patients and parents,^10,11^ indicating the need to find ways to help families prepare for their MDT appointment. While the prospect of meeting an MDT can provoke anxiety and anticipatory stress in parents, a patient information leaflet has been shown to reduce parents’ anxiety before their child's first craniofacial MDT appointment.^12^ Based on the findings of that study, a similar information leaflet was developed for the parents of children referred to the Norwegian craniofacial MDT. The aim was to evaluate the benefit of, and explore parents’ experience with, an information leaflet developed specifically for the parents of children referred to an outpatient MDT appointment.

## Methods

### Design

This study was conducted at the Centre for Rare Disorders at Oslo University Hospital as part of a larger project exploring the subjective experiences of patients with CFAs and their parents. The Data Protection Office at Oslo University Hospital granted ethical approval for the study (reference: 2016/14088). The study was partly funded by Foundation Dam (grant number: 2018/FO203158). The participants were informed about the study and provided written consent to participate.

The second and last authors developed a self-report questionnaire and an interview guide. The questionnaire was based on a previous study^12^ and included questions about the leaflet (with the response alternatives yes/no/unsure) and the option to add a free-text comment. Background information was also collected through the questionnaire. The interview guide (Supplemental Table 1) included a few questions about the parents’ experience of meeting the MDT (only for those participants who met the MDT for the first time), followed by more detailed questions about the leaflet. The aim of the interviews was not to generate in-depth data concerning parents’ experience of their child's treatment; rather, it was to elucidate how preliminary information about the MDT could help parents to feel prepared before attending the initial appointment. Given the study's aim, only data about the leaflet were included in the analysis.

### Leaflet Design and Distribution

The second author designed the leaflet in collaboration with the third and last authors. The leaflet featured an introduction to the MDT and its purpose, in addition to a group photo of the MDT members and a note setting out their names and roles. Furthermore, a general description was provided of the consultation room, the MDT appointment, and the examination of the child, as was advice on how to prepare the child for the appointment. There was also space for parents to write down any questions.

A draft of the leaflet was discussed with patient and parent representatives, the coordinators of the MDT, and counsellors at Centre for Rare Disorders. It was revised following their feedback. The final version of the leaflet was included with the appointment letter sent to all patients invited to the craniofacial MDT.

### Procedure

All the parents who attended an MDT appointment for the first time between August 2019 and December 2020 were invited to participate in the study. During a shorter period (August to December 2019), parents who had previous experience with the MDT were also invited to participate. This was done in order to ensure a sufficiently large sample for the quantitative data collection, since there is a very limited number of children coming to the MDT for a first appointment each year. The MDT coordinator asked potential participants at the day of their child's MDT appointment if they agreed to receive an invitation and information about the study. Those who accepted gave written consent, and were soon after contacted by one of the researchers. These potential participants received an invitation and information about the study, what participation would entail and key ethical considerations (confidentiality and right to withdraw), a questionnaire, and a new consent form, by post. Those who agreed to participate in the study could choose to complete the questionnaire only, or to complete the questionnaire and subsequently participate in a telephone interview. A member of the research team contacted those who agreed to be interviewed, and interviews were arranged as soon as possible.

The interviews were semi-structured and conducted by the second and last authors, both qualified clinical psychologists, as well as by a counsellor from Centre for Rare Disorders with a master's degree in educational and psychological counselling. None of the interviewers were part of the MDT. All the interviews were audio recorded and transcribed verbatim.

### Participants

#### Quantitative Data

The questionnaire was completed by 33 parents. The participants’ characteristics are summarised in Table 1. The participants confirmed their children to have the following diagnoses: craniofacial syndromes (e.g., Apert, Crouzon or mandibulofacial dysostosis with microcephaly, *n*  =  13), craniofacial microsomia (*n*  =  8) or another rare craniofacial condition (*n*  =  3, not specified to preserve anonymity). Nine children had a craniofacial condition that was not yet classified. The mean age of the children was 6.3 years (*SD*  =  5.0; range  =  4 months to 17 years).

**Table 1.** Participant Characteristics.

Participant characteristic	Questionnaire	Interview
Mean age (years)	36.6 (SD = 6.62; range = 23–48)	36.4 (SD = 7.47; range = 23–47)
		
	*n* (%)	*n* (%)
Gender		
Female	24 (72.7%)	12 (85.7%)
Male	9 (27.3%)	2 (14.3%)
Marital status		
Single	2 (6.1%)	1 (7.1%)
Cohabiting/married	31 (93.9%)	13 (92.9%)
Occupation requiring:		
Master's degree	10 (30.3%)	2 (14.3%)
Higher degree	17 (51.5%)	10 (71.4%)
Vocational training	4 (12.1%)	1 (7.1%)
Outside the labour force	2 (6.1%)	1 (7.1%)
Craniofacial MDT		
First time with MDT	16 (48.5%)	9 (64.3%)
Previously with MDT	17 (51.5%)	5 (35.7%)

*Note*. SD  =  standard deviation; MDT  =  multidisciplinary team. Thirty-three participants completed the questionnaire, fourteen of whom were interviewed.

#### Qualitative Data

A sub-sample of participants (*n*  =  14) agreed to participate in telephone interviews (see Table 1). Their children's mean age was 4.9 years (*SD*  =  4.3; range  =  4 months to 14 years). The distribution of craniofacial conditions was largely similar to that found in relation to the questionnaire data.

### Analysis

The quantitative data derived from the questionnaire were analysed using simple descriptive statistics (SPSS version 28, IBM Corp, Armonk, NY). The free-text comments were broadly categorised when possible or reported as stated, without quantification.

The qualitative data were analysed using a thematic, inductive data-driven approach drawn from Braun and Clarke.^13^ The first author read the interviews and wrote descriptive words or phrases next to associated excerpts in an iterative process and then, grouped codes and data extracts into themes. The thematic structure was reviewed and adjusted in dialogue with the last author.

## Results

### Questionnaire Data

No significant differences in responses were found between those who had previously met the craniofacial MDT and the first-time attendees. Therefore, the data were not subject to a group-wise analysis. The participants’ responses to each item are reported as frequencies in Table 2. Twenty-nine out of 33 participants used the free-text field one or more times.

**Table 2.** Participants’ Responses to Each Questionnaire Item.

	Response *n* (%)			No response *n* (%)
	Yes	No	Unsure	
Was the leaflet easy to understand?	33 (100%)	0 (0.0%)	0 (0.0%)	0 (0.0%)
Did you find the information in the leaflet to be helpful?	31 (93.9%)	0 (0.0%)	2 (6.1%)	0 (0.0%)
Did you find any information to be missing from the leaflet?	5 (15.2%)	19 (57.6%)	9 (27.2%)	0 (0.0%)
If you were worried about the appointment, did you find the leaflet helpful?	17 (51.5%)	1 (3.0%)	8 (24.2%)	7 (21.2%)
Did you find the leaflet to be helpful regarding the following questions and issues:				
The purpose of the appointment?	27 (81.8%)	1 (3.0%)	5 (15.2%)	0 (0.0%)
Why several people might be present during the appointment?	27 (81.8%)	3 (9.1%)	3 (9.1%)	0 (0.0%)
Who the people present might be?	30 (90.9%)	0 (0.0%)	3 (9.1%)	0 (0.0%)
How your child might be examined? (For example, measuring head circumference, checking dental braces.)	22 (66.7%)	2 (6.1%)	7 (21.2%)	2 (6.1%)
What questions you might be asked during the appointment?	22 (66.7%)	2 (6.1%)	8 (24.2%)	1 (3.0%)
The tip to write down questions you would like to ask?	26 (78.8%)	1 (3.0%)	4 (12.1%)	2 (6.1%)
The tip to prepare your child for the appointment?	28 (84.8%)	1 (3.0%)	3 (9.1%)	1 (3.0%)
If you had your first craniofacial MDT appointment after receiving the leaflet, please respond to these questions:				
Was it advantageous that you received the leaflet prior to the appointment?	15 (93.8%)	0 (0.0%)	1 (6.3%)	0 (0.0%)
Was it advantageous for your child that you received the leaflet prior to the appointment?	5 (31.3%)	3 (18.8%)	4 (25.0%)	4 (25%)

Thirty-three participants completed the questionnaire, although the last two questions were only completed by those participants who met the MDT for the first time (*n*  =  16).

#### General Views on the Leaflet

All the participants (*N*  =  33, 100%) found the leaflet easy to understand. Most participants (*n*  =  31, 93.9%) thought that it provided helpful information. There were comments in free-text about how the information had helped participants and the child to prepare for the appointment and manage their expectations.

Five participants (15.2%) thought there was information missing from the leaflet, while nine (27.2%) were unsure. Comments provided as free text specified what the participants felt was missing, for example, a more detailed description of the MDT setting, examples of questions to ask the specialists and advice on how to prepare the child for having their physical appearance discussed and evaluated during the appointment.

Seventeen participants (51.5%) found the leaflet helpful if they felt anxious about the appointment. There were free-text remarks from participants who had previously met the MDT about how they thought the leaflet would have helped manage their anxiety before their first visit. Free-text comments also included remarks about how the leaflet had given them a feeling of preparedness, or comments stating that that they had not felt anxious.

#### Views on the Description of the MDT Setting and Appointment

Most participants (*n*  =  27, 81.8%) found the leaflet helpful in explaining the appointment's purpose and why several specialists might be present. In free-text comments, participants remarked that the leaflet only covered the general purpose of the MDT. There were also free-text comments suggesting that the number of people who might be present should be stated more clearly in the leaflet. However, most participants (*n*  =  30, 90.9%) stated that the leaflet clearly described who the members of the MDT were.

Two-thirds of participants *(n*  =  22, 66.7%) found the leaflet helpful in explaining how their child might be examined and what questions might be asked during the appointment. The free-text comments included remarks about how the leaflet helped in preparing the child for the physical examination.

Twenty-six participants (78.8%) appreciated the tip to write down questions for the MDT, and most participants (*n*  =  28, 84.8%) found the tip about preparing their child helpful. However, there were free-text remarks about how, despite preparation, the MDT setting could still be perceived as unpredictable and emotionally uncomfortable. There were, also in free-text, comments mentioning that the child was too young to be prepared for the MDT.

#### Questions for the First-Time Attendees

Almost all the participants who attended the MDT for the first time (*n*  =  15, 93.8%) thought that it was advantageous to receive a leaflet before the appointment. The free-text comments included remarks about how the leaflet helped them to prepare, although they would have preferred to receive it closer to the appointment.

Views on whether the leaflet was advantageous for the child varied. Four participants (25.0%) were unsure about this issue and three participants (18.8%) answered ‘no’. There were free-text comments about how the leaflet had helped to start a conversation with the child about what would happen during the appointment.

### Qualitative Results

Three themes were identified in the interviews: Theme 1 describes the participants’ experience of the MDT appointment, Theme 2 presents some helpful aspects of the leaflet and Theme 3 captures the need for more information.

#### Theme 1. An Overwhelming Setting

The participants emphasised the importance of preparing to enter a room full of specialists and then having to sit in front of them. Some first-time attendees had not realised that all the MDT members would be present. Yet, some participants acknowledged that, even if they had read the information, the leaflet might not be sufficient to prepare for how it feels to enter the consultation room for the first time:

…when I first read the leaflet, it was completely okay. It was helpful information and all that. But it felt different once I got there. (Interview 2)

You cannot explain that on paper, what it feels like to enter a room with so many people who are there because of you. (Interview 14)

While the participants appreciated access to the MDT, some expressed unease about having their child examined by several specialists during the appointment, despite the leaflet including information about the assessment. Some also had concerns about the emotional and psychological impact on their child of having the child's appearance discussed in the consultation room. They wished the leaflet had included some information about this so that they could have prepared their child.

#### Theme 2. Reassuring Elements of the Leaflet

The participants were generally pleased to receive the information leaflet. Those who met the MTD for the first time thought they would have felt more anxious if they had not read it. Correspondingly, the participants who had attended appointments before stated that they would have liked to receive such information before their first appointment.

The descriptions of the consultation room and MDT setting were highlighted as key elements of the leaflet, although some participants would have preferred more detailed descriptions. The participants appreciated the advice about preparing their child for the appointment and writing down questions beforehand.

Because when it comes to disease or vulnerable situations … your memory might not be at its best… and you simply forget… and you have to ask the same things again. (Interview 12)

The participants described how the photo of the MDT members prepared them for who they would meet. For some, the photo suggested that many specialists would be present and interested in determining what their child needed. There were also remarks about how it was reassuring that the MDT members appeared approachable in the photo.

I think that just the fact that you get to see a photo of… what kind of crowd you will meet is very good. (…) Even if I couldn’t remember the faces, it was still good for my psyche. (Interview 5)

Some participants used the photo to prepare their child for the appointment and, later, to prompt a conversation about how it went. The photo was also useful when explaining the consultation to the child's siblings.

#### Theme 3. Desire for More Information and Predictability

The participants made several concrete suggestions for improvements to the leaflet and its distribution. One suggestion was to send the leaflet closer to the appointment date to make it easier to recollect the information on the day of the appointment. Another was to develop age-appropriate versions of the leaflet for children and include more advice on how to prepare the child.

I think that it [the leaflet] should… be just as much for the kids as it is for the adults. (Interview 8)

Other suggestions for changes to the information in the leaflet included providing an approximate number for how many specialists would be present at the appointment and specifying how long it would last. Additionally, it was considered helpful to provide information about potential additional examinations (eg, the child being photographed after the appointment) and recommend bringing copies of the child's medical records to the appointment.

The participants also proposed the inclusion of a variety of additional information relevant to their child in particular, for example, concerning a certain diagnosis or specific surgical procedure.

## Discussion

In the present study, the parents found the information leaflet about the MDT appointment to be helpful in preparing themselves and their child. Yet, many first-time attendees still found the MDT setting overwhelming, while some expressed the need for more detailed information prior to their visit in order to feel more prepared.

In line with a previous study,^12^ we found that the leaflet offered parents some predictability in an otherwise uncertain situation. However, some participants were unable to understand or benefit from all the information included in the leaflet, and some mentioned clarifications they felt would be important to add. Another finding was that the participants wanted more information specific to their child's individual treatment pathway. While the information leaflet will benefit from a revision based on the participants’ feedback, the findings also show that parents need comprehensive information and support related to their child's treatment and care, although their need for information and preparation probably extends beyond what a leaflet can offer. Unfortunately, the MDT setting will likely remain challenging for some, regardless of the extent of their preparations.

A study that investigated patients’ subjective experience of meeting with an MDT found that, although the participants appreciated the multidisciplinary and time-saving nature of the appointment, they felt uncomfortable or overwhelmed by both the setting and the number of specialists present,^10^ which is in line with the present results. As suggested in another study on this topic,^11^ MDTs should consider the number of specialists needed to be present during the appointment, and have one MDT member in charge of the dialogue with the family.

Despite information about the MDT being provided in the leaflet, some participants felt unprepared and unsettled during the first appointment. This emotional state may negatively affect parents’ involvement in shared decision-making or cause reluctance to request information from specialists during the appointment. As the reception of informational support and partnering in the care of the child are closely associated with parent empowerment, parents must be provided with conditions that make them feel comfortable enough to fully engage with the MDT appointment.^14^

A previous study showed that parents found it difficult to prepare their child for the craniofacial MDT when their own understanding of what lay ahead was limited.^11^ Research on multidisciplinary care for cleft lip and palate has shown that a coordinator, or a contact nurse, can be a valuable advocate and source of support for patients and parents when treatment and follow-up include a wide range of healthcare professionals.^15^ Based on the same concept, teams could consider designating a person as a point of contact between parents and the MDT. This person could help to introduce and explain the setting and aim of the MDT to parents before their first visit, so that parents may feel more prepared. Ideally, this person should also be present during the consultation, in order to support parents in their dialogue with the MDT, and check in with them after the appointment, in case there is a need for clarifications.

This study was a small-scale service evaluation among a rare population that was conducted within the boundaries of a clinical setting, meaning some limitations need to be considered. Ideally, participants would have received the leaflet closer to the MDT appointment to increase the likelihood of them recalling the information. Another consideration is that around half of the participants had been to the MDT before and consequently had previous knowledge about the setting. The gender distribution among the participants was uneven, especially in the interviews. The use of validated questionnaires would have been advantageous. Additionally, there was a lack of depth in the interview data, which allowed for only a very basic analysis of the parents’ reports.

Nevertheless, our findings indicate the importance of further research on the need for information and support among families of children with CFAs. It could be worthwhile to explore how the provision of written information prior to an MDT appointment could be combined with other preparatory methods, such as online support and the opportunity to talk about the MDT appointment with a member of staff before the visit.^16^

## Conclusions

Parents deemed a patient information leaflet helpful in preparing for their child's MDT appointment. The discomfort that some parents experience, particularly during the first meeting with the MDT, indicates the importance of support and information in connection with the child's MDT appointments and the MDT's follow-up of the child with CFA. Further research is needed to evaluate whether additional support tools, such as more detailed online support, and/or a dedicated coordinator, could help support parents by strengthening their understanding of the MDT’s role in their child's treatment pathway.

## Supplemental Material

sj-docx-1-cpc-10.1177_10556656231219579 - Supplemental material for Parents’ Perception of the Benefit of Receiving a Patient Information Leaflet Prior to Attending a Craniofacial Multidisciplinary Team Appointment Supplemental material, sj-docx-1-cpc-10.1177_10556656231219579 for Parents’ Perception of the Benefit of Receiving a Patient Information Leaflet Prior to Attending a Craniofacial Multidisciplinary Team Appointment by Moa Millgård, Anita Myhre, Bernt J. Due-Tønnessen and Kristin J. Billaud Feragen in The Cleft Palate Craniofacial Journal

## References

[1] Tang AR Chen JW Sellyn GE , et al. Evaluating caregiver stress in craniosynostosis patients. J Neurosurg Pediatr. 2022;30(2):1–8. doi:10.3171/2022.4.Peds21596 35561696

[2] Costa B Edwards W Wilkinson-Bell K Stock NM . Raising a child with craniosynostosis: Psychosocial adjustment in caregivers. Cleft Palate Craniofac J. 2023;60(10):1284–1297. doi:10.1177/10556656221102043 35786018

[3] Feragen KJB Myhre A Stock NM . “Will you still feel beautiful when you find out you are different?”: Parents’ experiences, reflections, and appearance-focused conversations about their child’s visible difference. Qual Health Res. 2022;32(1):3–15. doi:10.1177/10497323211039205 34596475 PMC8739583

[4] Myhre A Råbu M Feragen KJB . The need to belong: Subjective experiences of living with craniofacial conditions and undergoing appearance-altering surgery. Body Image. 2021;38:334–345. doi:10.1016/j.bodyim.2021.05.008 34087543

[5] Feragen KB Stock NM Myhre A Due-Tønnessen BJ . Medical stress reactions and personal growth in parents of children with a rare craniofacial condition. Cleft Palate Craniofac J. 2020;57(2):228–237. doi:10.1177/1055665619869146 31426676

[6] Lim J Davis A Tang AR Shannon CN Bonfield CM . Caregiver stress in children with craniosynostosis: A systematic literature review. Childs Nerv Syst. 2019;35(2):217–225. doi:10.1007/s00381-018-3959-7 30155782

[7] Gallagher ER Fulton GK Susarla SM Birgfeld CB . Multidisciplinary care considerations for patients with craniosynostosis. Oral Maxillofac Surg Clin North Am. 2022;34(3):353–365. doi:10.1016/j.coms.2022.04.001 35787826

[8] Buchanan EP Xue Y Xue AS Olshinka A Lam S . Multidisciplinary care of craniosynostosis. J Multidiscip Healthc. 2017;10:263–270. doi:10.2147/jmdh.S100248 28740400 PMC5505551

[9] Mathijssen IMJ ; Working Group Guideline Craniosynostosis. Updated guideline on treatment and management of craniosynostosis. J Craniofac Surg. 2021;32(1):371–450. doi:10.1097/SCS.0000000000007035 33156164 PMC7769187

[10] Myhre A Agai M Dundas I Feragen KB . “All eyes on me”: A qualitative study of parent and patient experiences of multidisciplinary care in craniofacial conditions. Cleft Palate Craniofac J. 2019;56(9):1187–1194. doi:10.1177/1055665619842730 31010312

[11] Feragen K Myhre A Stock NM . “Exposed and vulnerable”: Parent reports of their child's experience of multidisciplinary craniofacial consultations. Cleft Palate Craniofac J. 2019;56(9):1230–1238. doi:10.1177/1055665619851650 31142141

[12] Pidgeon TE Blore CD Webb Y Horton J Evans M . A patient information leaflet reduces parental anxiety before their child's first craniofacial multidisciplinary outpatient appointment. J Craniofac Surg. 2017;28(7):1772–1776. doi:10.1097/scs.0000000000003955 28891904

[13] Braun V Clarke V . Using thematic analysis in psychology. Qual Res Psychol. 2006;3(2):77–101. doi:10.1191/1478088706qp063oa

[14] Ashcraft LE Asato M Houtrow AJ Kavalieratos D Miller E Ray KN . Parent empowerment in pediatric healthcare settings: A systematic review of observational studies. Patient. 2019;12(2):199–212. doi:10.1007/s40271-018-0336-2 30328069 PMC6397702

[15] Feragen KB Rumsey N Heliövaara A , et al. Scandcleft randomised trials of primary surgery for unilateral cleft lip and palate: 9. Parental report of social and emotional experiences related to their 5-year-old child’s cleft diagnosis. J Plast Surg Hand Surg. 2017;51(1):73–80. doi:10.1080/2000656X.2016.1254643 28218553

[16] Kerimaa H Ruotsalainen H Kyngäs H Miettunen J Pölkki T . Effectiveness of interventions used to prepare preschool children and their parents for day surgery: A systematic review and meta-analysis of randomised controlled trials. J Clin Nurs. 2023;32(9-10):1705–1722. doi:10.1111/jocn.16156 34870345

