# Supplementary material for: Parents’ Perception of the Benefit of Receiving a Patient Information Leaflet Prior to Attending a Craniofacial Multidisciplinary Team Appointment
Source: Cleft Palate Craniofac J. 2023 Dec 13;62(3):527–33. doi: 10.1177/10556656231219579 (PMC11969871; doi:10.1177/10556656231219579)
Supplement: sj-docx-1-cpc-10.1177_10556656231219579 - Supplemental material for Parents’ Perception of the Benefit of Receiving a Patient Information Leaflet Prior to Attending a Craniofacial Multidisciplinary Team Appointment [file sj-docx-1-cpc-10.1177_10556656231219579.docx]

**Supplemental Table 1.** Interview guide.

**Questions for all interviewees about the patient information leaflet:**

You received a leaflet with the craniofacial MDT appointment letter. Do you remember the leaflet?

How was it to read the leaflet? (What kinds of thoughts and feelings did you have when you read the leaflet?)

What kinds of expectations did you have when you read the leaflet?

Did the MDT appointment meet your expectations?

In the leaflet, there was a tip about how it might be wise to prepare your child for the appointment. Was it helpful? (Prompt to elaborate.) Why and how did you use that tip or why was it not helpful?

Do you think anything else should have been included in the leaflet?

**Additional questions for interviewees who met the MDT for the first time:**

If you were worried about the appointment, did you find the leaflet helpful? (Prompt to elaborate.)

Now that you have met with the MDT, do you think there was any information missing from the leaflet? (If yes, prompt to elaborate.)

Do you think meeting the MDT would have been different in any way if you had not read the leaflet first?

Do you feel that the leaflet prepared you for the appointment?

**Additional question for interviewees who had met the MDT on at least two occasions:**

Do you think your very first meeting with the MDT would have been different in any way if you had received the leaflet prior to that appointment?

**Questions for all interviewees about the leaflet design:**

What do you think about the language used in the leaflet? (Easy/hard to understand, etc.)

What do you think about the photo and the text underneath the photo? (Photo of the MDT members.)

Was there a suitable amount of text or should there be more/less? (Prompt to elaborate.)

Do you have any other comments about the leaflet?
